# Comparing the Clinical Utility and Diagnostic Performance of CSF P-Tau181, P-Tau217, and P-Tau231 Assays

**DOI:** 10.1212/WNL.0000000000012727

**Published:** 2021-10-26

**Authors:** Antoine Leuzy, Shorena Janelidze, Niklas Mattsson-Carlgren, Sebastian Palmqvist, Dirk Jacobs, Claudia Cicognola, Erik Stomrud, Eugeen Vanmechelen, Jeffrey L. Dage, Oskar Hansson

**Affiliations:** From the Clinical Memory Research Unit (A.L., S.J., N.M.-C., S.P., C.C., E.S., O.H.), Department of Clinical Sciences, Lund University, Malmö; Department of Neurology (N.M.-C.) and Memory Clinic (S.P., E.S., O.H.), Skåne University Hospital, Lund; Wallenberg Centre for Molecular Medicine (N.M.-C.), Lund University, Sweden; ADx NeuroSciences NV (D.J., E.V.), Ghent, Belgium; and Eli Lilly and Company (J.L.D.), Indianapolis, IN.

## Abstract

**Background and Objectives:**

Phosphorylated tau (p-tau) in CSF is considered an important biomarker in Alzheimer disease (AD) and has been incorporated in recent diagnostic criteria. Several variants exist, including p-tau at threonines 181 (p-tau181), 217 (p-tau217), and 231 (p-tau231). However, no studies have compared their diagnostic performance or association to β-amyloid (Aβ) and tau-PET. Understanding which p-tau variant to use remains an important yet answered question. We aimed to compare the diagnostic accuracy of p-tau181, p-tau217, and p-tau231 in CSF for AD and their association with Aβ and tau-PET.

**Methods:**

A total of 629 participants in the Swedish BioFINDER-2 study were included (cognitively unimpaired, n = 334; Aβ-positive mild cognitive impairment, n = 84; AD dementia, n = 119; and non-AD disorders, n = 92). In addition to p-tau181 and p-tau217 measured using assays with the same detector antibodies from Eli Lilly (p-tau181_Lilly_, p-tau217_Lilly_) and p-tau231, we also included p-tau181 measurements from 2 commonly used assays (Innotest and Elecsys).

**Results:**

Although all p-tau variants increased across the AD continuum, p-tau217_Lilly_ showed the greatest dynamic range (13-fold increase vs 1.9–5.4-fold increase for other p-tau variants for AD dementia vs non-AD). P-Tau217_Lilly_ showed stronger correlations with Aβ- and tau-PET (*p* < 0.0001). P-Tau217_Lilly_ exhibited higher accuracy than other p-tau variants for separating AD dementia from non-AD (area under the curve [AUC], 0.98 vs 0.88 [*p* < 0.0001] - 0.96 [*p* < 0.05]) and for identifying Aβ-PET (AUC, 0.86 vs 0.74 [*p* < 0.0001] and 0.83 [*p* < 0.001]) and tau-PET positivity (AUC, 0.94 vs 0.80—0.92, *p* < 0.0001). Finally, p-Tau181_Lilly_ generally performed better than the other p-tau181 assays (e.g., AD dementia vs non-AD, AUC, 0.96 vs 0.88 [p-tau181_Innotest_] and 0.89 [p-tau181_Elecsys_]; *p* < 0.0001).

**Discussion:**

CSF p-tau217_Lilly_ seems to be more useful than other included p-tau assays in the workup of AD. Varied results across p-tau181 assays highlights the importance of anti-tau antibodies for biomarker performance.

**Classification of Evidence:**

This study provides Class II evidence that p-tau217 provides higher diagnostic accuracy for diagnosis of AD dementia than p-tau181 or p-tau231.

In addition to extracellular deposition of β-amyloid (Aβ) plaques, Alzheimer disease (AD) is defined by the intracellular aggregation of tau in neurofibrillary tangles (NFTs), composed of abnormally hyperphosphorylated tau.^1^ Tau pathology is thought to be reflected in CSF levels of phosphorylated tau (p-tau). CSF p-tau has shown high prognostic accuracy for AD and for predicting cognitive decline in cognitively unimpaired (CU) individuals and in patients with mild cognitive impairment (MCI) due to AD.^2,3^ As CSF p-tau levels are higher in AD compared to other non-AD neurodegenerative disorders, including progressive supranuclear palsy (PSP), corticobasal syndrome (CBS), frontotemporal dementia (FTD), and vascular dementia (VaD), it has also proven of use in the differential diagnosis of AD vs other dementias.^4^

Tau in CSF is largely present in the form of different fragments.^5-8^ Of these, N-terminal and midregion variants are the most abundant. In addition, numerous sites exist where tau can undergo abnormal hyperphosphorylation.^9^ The most commonly used assays for p-tau, however, use antibodies targeting the midregion of tau as well as an antibody targeting tau phosphorylated at threonine-181 (p-tau181).^10^ Besides p-tau181, increased levels of mid tau fragments phosphorylated at threonine-231 (p-tau231) appear to be an early occurrence in AD, preceding the formation of paired helical filaments.^11^ Although studies have shown that p-tau231 can accurately discriminate patients with AD from CU individuals and patients with non-AD disorders, similar to p-tau181, a series of postmortem studies that examined both measures reported that CSF p-tau231 was better associated with neocortical fibrillary pathology than CSF p-tau181.^12,13^ Recently, p-tau fragments phosphorylated at threonine-217 (p-tau217) were also measured in CSF.^14^ Compared to p-tau181, p-tau217 showed stronger correlations with Aβ and tau-PET and more accurately distinguished AD dementia from non-AD neurodegenerative disorders.^14^ Additional work has shown that p-tau181 and p-tau217 are increased already in preclinical AD (Aβ-positive CU), with these increases preceding tau-PET positivity and even occurring prior to the threshold for Aβ-PET positivity.^15,16^

Overall, findings indicate that increases in CSF p-tau occur in response to very early Aβ pathology and precede widespread tau aggregation. Thus far, however, there are no studies comparing p-tau181, p-tau217, and p-tau231 levels in relation to Aβ and tau-PET across the symptomatic stages of AD, nor data directly comparing their diagnostic performance for separating AD dementia from non-AD neurodegenerative disorders and for identifying abnormal Aβ and tau-PET status. Because CSF p-tau is an important biomarker in the workup for AD and is incorporated in its diagnostic criteria,^17^ it is of great importance to determine which of these p-tau variants to use, especially as clinical heterogeneity and different stages in AD may be determined by heterogeneity in the post-translational modification (PTM) of tau.^18^

We aimed to address these questions using cross-sectional data from a well-characterized cohort, ranging from Aβ-negative CU individuals to Aβ-positive CU individuals and Aβ-positive patients with MCI or AD dementia. In addition to comparing p-tau181 and p-tau217 measured using assays with the same detector antibodies from Eli Lilly (p-tau181_Lilly_ and p-tau217_Lilly_) with p-tau231 measured using an assay with a phospho-specific cis-conformational monoclonal antibody (p-tau231_ADx_), we also compared p-tau181_Lilly_ with p-tau181 measurements from 2 commonly used assays (Innotest[p-tau181_Innotest_] and Elecsys[p-tau181_Elecsys_]).

## Methods

### Participants

We included participants from the prospective and longitudinal Swedish BioFINDER-2 study (clinical trial NCT03174938), including CU participants and patients with MCI, AD dementia, and non-AD neurodegenerative disorders. CU individuals were aged ≥60 years and did not have MCI or dementia.^17^ Patients with MCI fulfilled DSM-5 criteria for mild neurocognitive disorder^19^ and patients with AD dementia fulfilled the DSM-5 criteria for major neurocognitive disorder due to AD.^19^ Patients with non-AD disorders fulfilled diagnostic criteria for PSP or CBS,^20,21^ Parkinson disease (PD) with or without cognitive impairment,^22^ FTD,^23^ or VaD.^24^ Further details pertaining to inclusion and exclusion criteria are described in the Supplement (eAppendix 1, available from Dryad, doi.org/10.5061/dryad.4f4qrfjc7). Groups were established without the use of biomarkers, but CU and MCI participants were subdivided based on Aβ status, determined using CSF Aβ42/Aβ40 (Innotest; Fujirebio) and a cutoff of <0.089.^25^ We included only Aβ-positive AD dementia cases, in keeping with the research framework by the National Institute on Aging–Alzheimer's Association.^17^ As Aβ-PET is by design performed only in CU individuals and in patients with MCI, CSF Aβ42/Aβ40 was thus chosen to have a common measure of Aβ pathology across all participants.

### Standard Protocol Approvals, Registrations, and Patient Consents

All participants gave written informed consent. Ethical approval was given by the Regional Ethical Committee in Lund, Sweden. Approval for PET imaging was obtained from the Swedish Medical Products Agency and the local Radiation Safety Committee at Skåne University Hospital, Sweden.

### CSF P-Tau181 and P-Tau217 Measurements (Eli Lilly)

Analysis of CSF mid-domain p-tau181_Lilly_ and p-tau217_Lilly_ was performed at Eli Lilly and Company using the Meso Scale Discovery (MSD) platform, as previously described.^14^ The anti-p-tau217 antibody IBA413 and anti-p-tau181 antibody AT270 were used as capture antibodies in the p-tau181 and p-tau217 assays, respectively. Capture antibodies were conjugated with biotin (Thermo Scientific). Sulfo-tag (MSD) conjugated LRL antibody was used as a detector in both assays. The assays were calibrated using a recombinant tau (4R2N) protein that was phosphorylated in vitro using a reaction with glycogen synthase kinase-3 and characterized by mass spectrometry. Samples were analyzed in duplicate and the mean of duplicates was used in statistical analysis. Ten samples from Aβ-negative CU individuals (1.59%) were below the limit of detection and were excluded, as were 5 patients with AD with very high p-tau217_Lilly_ levels (>3 SD above the mean).

### CSF P-Tau231 Measurements (ADx NeuroSciences)

CSF p-tau231_ADx_ was measured at ADx NeuroSciences with a research sandwich ELISA (version 1) according to the kit instructions. Phospho-specific cis-conformational monoclonal antibody ADx253 (T1H11) was used as a capture antibody and biotinylated pan-tau monoclonal antibody ADx205 (epitope region aa224-238) as a detector. The assay was calibrated using an in-house designed synthetic peptide combining both antibody epitopes and having the corresponding threonine231 phosphorylated and proline232 replaced by a homoproline, Pip, to reflect cis selectivity of ADx253. In all prior analyses, we observed a consistent low interplate variability well below 15%. Because p-tau231 quantifications require 80 mL per single measurements—requiring at least 160 mL per result—we opted to run the p-tau231 measurement in singlicate as the study was designed to explore the difference between the phospho-tau assays. Quality control samples run on each plate, which were leftovers of CSF, confirmed high precision of these runs with coefficients of variation below 15%.

### CSF P-Tau181 Measurements (Innotest and Elecsys)

For comparative purposes, we also included p-tau181 measured using the well-known commercially available ELISA from Innotest (Fujirebio) (p-tau181_Innotest_)^10^ and the fully automated Elecsys electrochemiluminescence immunoassay (Roche Diagnostics) (p-tau181_Elecsys_) on a cobas e601 analyzer (software version 05.02).^26^ For p-tau181_Innotest_, monoclonal capture/detection antibodies were HT7 (epitope region aa159-162) and AT270. For p-tau181_Elecsys_, a biotinylated monoclonal antibody specific for phosphorylation at threonine 181 (11H5V1) and a monoclonal tau-specific antibody (PC1C6) were used (epitope region aa195-202). All samples were analyzed at the Clinical Neurochemistry Laboratory in Mölndal, Sweden.

### Image Acquisition and Processing

Aβ and tau-PET were performed using [^18^F]flutemetamol and [^18^F]RO948, respectively, as described elsewhere.^27,28^ Briefly, dynamic (list-mode) studies were performed over the 90- to 100-minute postinjection interval for [^18^F]flutemetamol and the 70- to 90-minute interval for [^18^F]RO948. Standardized uptake value ratio (SUVR) images were created using the pons ([^18^F]flutemetamol) and inferior cerebellar cortex ([^18^F]RO948) as reference regions. A high-resolution T1-weighted MRI was performed using a Siemens-3T MAGNETOM Prisma scanner for PET image coregistration and template normalization.

### Regions of Interest and Cutoffs

Target regions of interest (ROIs) were chosen on the basis of previously published findings: a neocortical meta-ROI for Aβ-PET (prefrontal, lateral temporal, parietal, anterior cingulate, and posterior cingulate/precuneus)^28,29^ and, for tau-PET, the entorhinal cortex (Braak I/II), a temporal meta-ROI (amygdala, inferior/middle temporal gyri, fusiform gyrus, and parahippocampal gyrus, approximating Braak III/IV),^30^ and a neocortical meta-ROI capturing late stage tau pathology (Braak V/VI).^31^ A priori cutoffs based on Gaussian mixture modeling (Aβ-PET)^14^ and the mean SUVR within a given ROI plus 2.5 SDs among young Aβ-negative CU individuals (tau-PET)^25^ were used to define positivity within these ROIs.

### Statistical Analyses

Group differences in age-adjusted CSF p-tau levels were assessed using pairwise analysis of variance–based comparisons of linear regression models. Associations between CSF p-tau isoforms and between p-tau isoforms and ROI-based Aβ and tau-PET SUVR values were assessed using correlation analysis; differences between correlation coefficients were tested using a confidence interval (CI)–based approach with bootstrapping.^32^ Log-transformed biomarker and PET measures were used in regression analyses. Generalized additive models with cubic regression splines were used to compare the slopes of CSF p-tau isoforms (mean change from Aβ-negative CU) across different tau and Aβ-PET SUVR values. Differences between the estimated functions were assessed by means of bootstrapped CIs. These were computed by repeatedly (n = 10,000) resampling the dataset (with replacement) and calculating the differences between spline fits. The discriminative performance of CSF p-tau measures was assessed using the area under the receiver operating characteristic (ROC) curve (AUC), adjusted for age. Significant differences in AUC values were tested using DeLong statistics^33^ and Bonferroni correction was applied to account for multiple comparisons. In addition to AUC, sensitivity and specificity at the cutoff that resulted in the highest Youden index (sensitivity + specificity – 1) are reported. Analyses were performed in R, v.4.0.2, with significance set at *p* < 0.05, 2-tailed. Voxelwise analyses examining the association between CSF p-tau levels and Aβ and tau-PET were performed using multilinear models, as implemented in SPM12, adjusted for age and the interval between lumbar puncture and PET scan.

### Data Availability

Anonymized study data for the primary analyses presented in this report are available on request from any qualified investigator for purposes of replicating the results.

## Results

### Participants

We included 629 participants, including 334 CU controls (253 [76%] Aβ-negative and 81 [24%] Aβ-positive), 84 Aβ-positive MCI, 119 Aβ-positive AD dementia, and 92 with a non-AD neurodegenerative disorder (21 FTD, 40 PD with or without cognitive impairment, 20 PSP/CBD, and 11 VaD; overall, 15% [n = 14] showed Aβ positivity). Demographic and clinical characteristics are summarized in Table 1. For a flow diagram of participants included in the study, see eFigure 1 (available from Dryad, doi.org/10.5061/dryad.4f4qrfjc7).

**Table 1:**
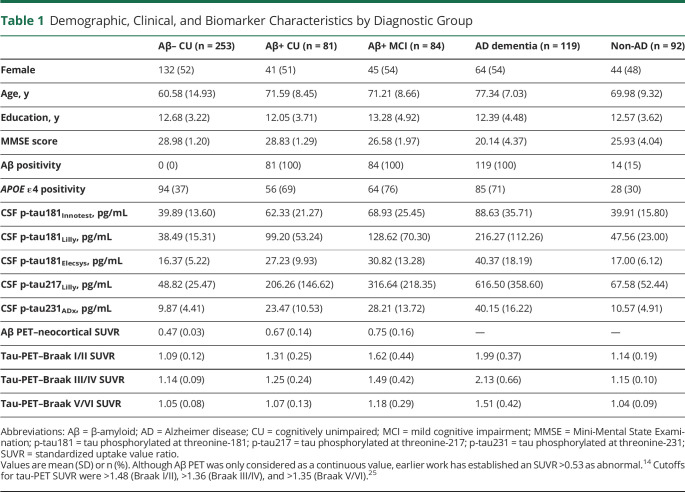
Demographic, Clinical, and Biomarker Characteristics by Diagnostic Group

### Correlations Between P-Tau Isoforms

**Figure 1:**
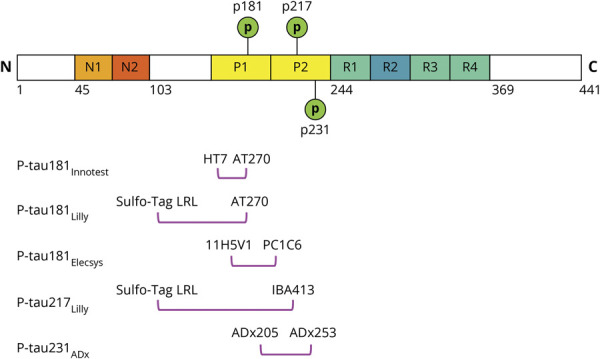
Schematic Overview of the Included Phosphorylated Tau (P-Tau) Assays Schematic illustration of full-length tau441, including N-terminal, proline-rich region, microtubuli binding domain, and C-terminal. Anti-tau antibodies are indicated for each of the 5 included p-tau assays under the respective epitope region.

A schematic overview of the included p-tau assays is provided in Figure 1. P-Tau isoforms were strongly correlated across all participants (range 0.853–0.977, all *p <* 0.0001) (eFigure 2, available from Dryad, doi.org/10.5061/dryad.4f4qrfjc7). These associations were significant in Aβ-positive CU, Aβ-positive MCI, and AD dementia, but not in Aβ-negative individuals. As moderate but significant correlations were observed between age and CSF p-tau levels (eTables 1 and 2, available from Dryad, doi.org/10.5061/dryad.4f4qrfjc7), age was accounted for when comparing CSF p-tau levels across tau-PET–based Braak stages and diagnostic groups.

### Correlations Between CSF P-Tau and Aβ and Tau-PET

Correlations between CSF p-tau isoforms and Aβ and tau-PET SUVR values in Braak ROIs are reported in the Supplement (eTable 3, available from Dryad, doi.org/10.5061/dryad.4f4qrfjc7). Correlations between the CSF p-tau species and Aβ or tau-PET did not differ significantly by *APOE* status (ε4 carrier vs noncarrier), age (over/under median split age [70 years]), or sex (male vs female) (data not shown). Using Aβ-PET, p-tau217_Lilly_ showed the strongest correlation with neocortical SUVR values in CU individuals (*r* = 0.789, *p* < 0.001). This correlation was significantly higher than those for p-tau181_Innotest_ (*r* = 0.497), p-tau181_Lilly_ (*r* = 0.737), p-tau181_Elecsys_ (*r* = 0.581), and p-tau231_ADx_ (*r* = 0.724) (*p* < 0.0001). In Aβ-positive MCI, p-tau217_Lilly_ also showed the strongest correlation (*r* = 0.516, *p* < 0.001) with Aβ-PET; this correlation was significantly stronger than those for p-tau181_Innotest_ (*r* = 0.312) and p-tau181_Elecsys_ (*r* = 0.314) (*p* < 0.001). Findings from voxel-wise analyses were consistent with these ROI-based results and also highlighted the stronger correlations between p-tau isoforms and Aβ-PET in CU individuals (eFigure 3, available from Dryad, doi.org/10.5061/dryad.4f4qrfjc7). When correlation analyses were repeated in CU individuals by Aβ status, significant associations between CSF p-tau measures and Aβ-PET were found only in the Aβ-positive CU group (eTable 4, available from Dryad, doi.org/10.5061/dryad.4f4qrfjc7).

Using tau-PET, CSF p-tau217_Lilly_ was most strongly associated p-tau variant in CU participants, with the strongest correlation seen in the Braak I/II ROI (*r* = 0.683). This correlation was significantly higher than those for p-tau181_Innotest_ (*r* = 0.485) (*p* < 0.0001), p-tau181_Lilly_ (*r* = 0.640) (*p* < 0.001), p-tau181_Elecsys_ (*r* = 0.546) (*p* < 0.0001), and p-tau231_ADx_ (*r* = 0.605) (*p* < 0.001). A similar pattern was seen when looking at Braak III/IV and V/VI.

In Aβ-positive cognitively impaired individuals (i.e., Aβ-positive MCI and AD dementia), p-tau217_Lilly_ showed the highest correlation to tau-PET in the Braak III/IV ROI (*r* = 0.592); this association was significantly higher than for p-tau181_Innotest_ (*r* = 0.272) (*p* < 0.0001), p-tau181_Lilly_ (*r* = 0.486) (*p* < 0.0001), p-tau181_Elecsys_ (*r* = 0.301) (*p* < 0.001), and p-tau231_ADx_ (*r* = 0.393) (*p* < 0.0001). This pattern also held when looking at Braak I/II and V/VI ROIs. In addition, p-tau181_Lilly_ showed significantly higher correlations with tau-PET across all Braak ROIs as compared to p-tau181_Innotest_, p-_tau181_Elecsys_, and p-tau231_ADx_. Voxelwise analyses (eFigure 4) supported these findings and in particular highlighted the stronger associations of p-tau217_Lilly_ to tau-PET when compared to the associations between tau-PET and other p-tau variants. Similar to findings using Aβ-PET, associations between CSF p-tau measures and tau-PET were significant only in the Aβ-positive CU group (eTable 4, available from Dryad, doi.org/10.5061/dryad.4f4qrfjc7) when repeating analyses by Aβ status.

### CSF P-Tau Slopes as a Function of Aβ and Tau-PET

**Figure 2:**
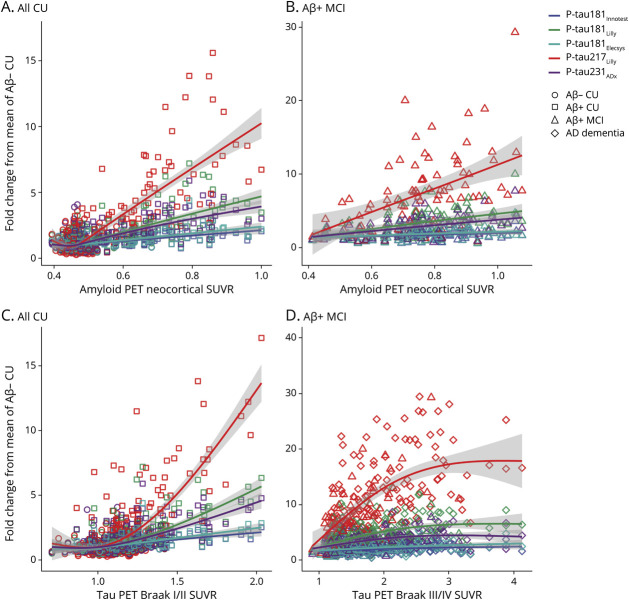
CSF Phosphorylated Tau (P-Tau) Slopes as a Function of β-Amyloid (Aβ) and Tau-PET Standardized Uptake Value Ratio (SUVR) CSF p-tau levels (expressed as mean fold change relative to the mean of Aβ-negative cognitively unimpaired [CU] participants) are shown against global Aβ PET neocortical SUVR across all CU participants (A) and in Aβ-positive mild cognitive impairment (MCI) (B). Panels C and D show corresponding plots for tau-PET in all CU participants (Braak I/II) and in all Aβ-positive cognitively impaired participants (Braak III/IV) (i.e., Aβ-positive MCI and Alzheimer disease [AD] dementia combined). Generalized additive models with cubic regression splines were used to compare the slopes of CSF p-tau isoforms across different Aβ and tau-PET SUVR values. Shaded gray areas indicate 95% confidence intervals.

Spline models examining CSF p-tau concentrations across Aβ and tau-PET are shown in Figure 2; CIs for differences in p-tau biomarkers at specified SUVR values are detailed in the Supplement (eTable 5, available from Dryad, doi.org/10.5061/dryad.4f4qrfjc7). Using Aβ–PET, the slope of p-tau217_Lilly_ was significantly different from those of p-tau181_Innotest_, p-tau181_Lilly_, p-tau181_Elecsys_, and p-tau231_ADx_ in Aβ-positive CU. The slope of p-tau181_Lilly_ differed significantly from those of p-tau181_Innotest_ and p-tau181_Elecsys_; no significant difference was seen between CIs for p-tau181_Lilly_ and p-tau231_ADx_, however. The same pattern was seen for p-tau217_Lilly_ when looking at Aβ-PET in Aβ-positive MCI. When analyses were performed separately in Aβ-positive and Aβ-negative CU individuals (eFigure 5, eTable 6, available from Dryad, doi.org/10.5061/dryad.4f4qrfjc7), increasing fold change with increasing SUVR values and separation of p-tau trajectories was largely confined to the Aβ-positive CU group.

Using tau-PET SUVR in the Braak I/II ROI in CU individuals, the slope of p-tau217_Lilly_ differed significantly from those of p-tau181_Innotest_, p-tau181_Lilly_, p-tau181_Elecsys_, and p-tau231_ADx_ at SUVR values of 1.5 or greater. The slopes of p-tau181_Lilly_ and p-tau231_ADx_ also differed significantly from those of p-tau181_Innotest_ and p-tau181_Elecsys_; no significant difference was seen between p-tau181_Lilly_ and p-tau231_ADx_. The same pattern was seen when using the Braak III/IV ROI in Aβ-positive cognitively impaired participants and using the Braak V/VI ROI (data not shown). Similar to the analyses with Aβ-PET, greater fold change at higher SUVR levels and separation of p-tau trajectories was largely confined to the Aβ-positive CU group (eFigure 5 and eTable 6, available from Dryad, doi.org/10.5061/dryad.4f4qrfjc7).

### CSF P-Tau Levels by Tau-PET–Based Braak Stages

**Figure 3:**
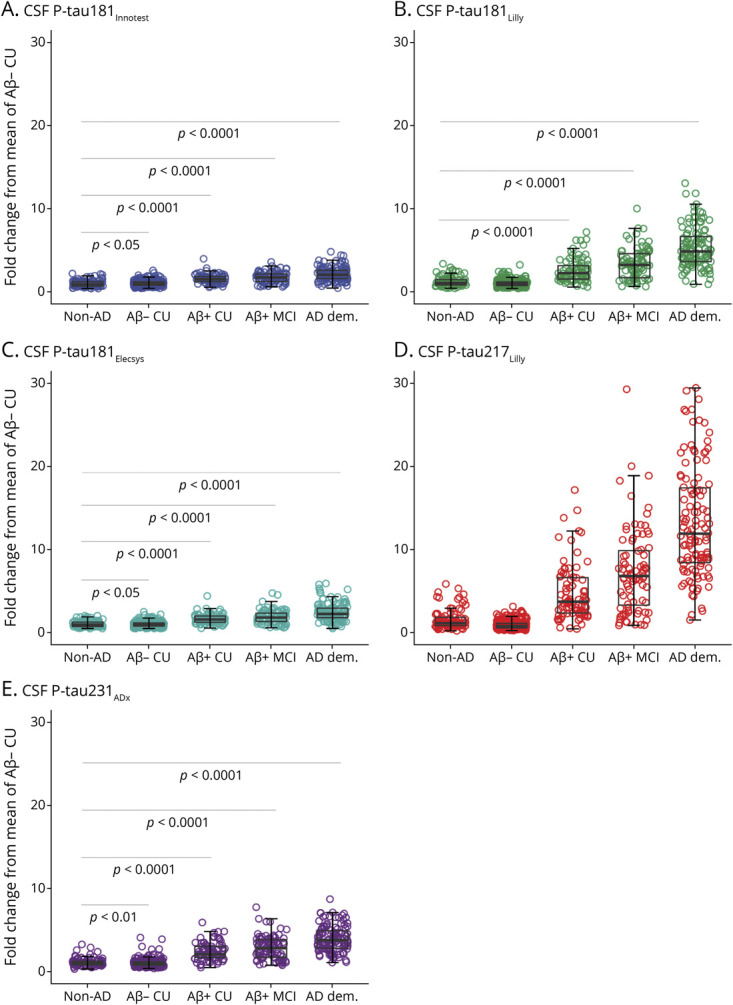
CSF Phosphorylated Tau (P-Tau) Across PET-Based Braak Stages Levels of CSF p-tau181_Innotest_ (A), p-tau181_Lilly_ (B), p-tau181_Elecsys_ (C), p-tau217_Lilly_ (D), and p-tau231_ADx_ (E) are expressed relative to the mean of participants showing no abnormal tau-PET standardized uptake value ratio (SUVR) values in any of the investigated regions of interest (Braak 0, n = 437). Tau positivity in Braak stages III/VI was established using a priori cutoffs based on the mean SUVR within a given region of interest plus 2.5 SD among β-amyloid (Aβ)–negative young controls. Solid gray horizontal lines indicate age-adjusted group comparisons: Alzheimer disease (AD) dementia higher than all groups (*p* < 0.001); Aβ-positive mild cognitive impairment (MCI) higher than cognitively unimpaired (CU) and non-AD (*p* < 0.001); Aβ-positive CU higher than Aβ-negative CU and non-AD (*p* < 0.001). In order to facilitate comparison between p-tau measures, y-axes were scaled to the maximum fold change seen across biomarkers. AD dem. = Alzheimer disease dementia; non-AD = non-Alzheimer disease neurodegenerative disorders.

When dividing participants on the basis of their tau-PET status in Braak ROIs (Figure 3) (i.e., [^18^F]RO948 negative [Braak 0] or abnormal retention in the Braak I/II ROI only or abnormal retention in the Braak III/IV ROI [but not V/VI] or Braak V/VI), fold change (relative to the mean of the Braak 0 group) was highest for p-tau217_Lilly_ (Braak III/IV, 4.69 [95% CI, 4.15–5.24]; V/VI, 6.93 [95% CI, 6.21–7.65]) followed by p-tau181_Lilly_ (Braak III/IV, 2.93 [95% CI, 2.63–3.23]; V/VI, 3.78 [95% CI, 3.41–4.15]), p-tau231_ADx_ (Braak III/IV, 2.39 [95% CI, 2.19–2.59]; V/VI, 2.93 [95% CI, 2.66–3.20]), p-tau181_Elecsys_ (Braak III/IV, 1.88 [95% CI, 1.71–2.04]; Braak V/VI, 2.23 [95% CI, 2.04–2.44]), and p-tau181_Innotest_ (Braak III/IV, 1.71 [95% CI, 1.57–1.85]; Braak V/VI, 1.98 [95% CI, 1.82–2.14]).

### CSF P-Tau Levels by Diagnostic Group

**Figure 4:**
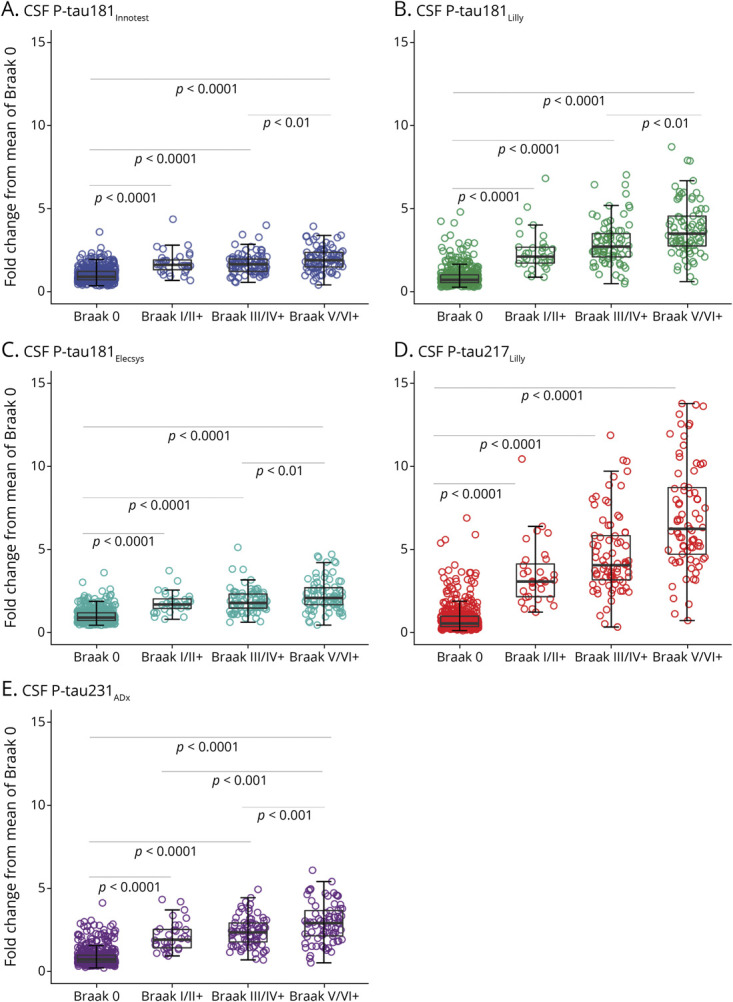
CSF Phosphorylated Tau (P-Tau) Across Diagnostic Groups Levels of CSF p-tau181_Innotest_ (A), p-tau181_Lilly_ (B), p-tau181_Elecsys_ (C), p-tau217_Lilly_ (D), and p-tau231_ADx_ (E) are expressed relative to the mean of β-amyloid (Aβ)–negative participants (n = 253). Solid gray horizontal lines indicate age-adjusted group comparisons: Alzheimer disease (AD) dementia higher than all groups (*p* < 0.001); Aβ-positive mild cognitive impairment higher than cognitively unimpaired (CU) and non-AD neurodegenerative disorders (*p* < 0.001); Aβ-positive CU higher than Aβ-negative CU and non-AD (*p* < 0.001). In order to facilitate comparison between p-tau measures, y-axes were scaled to the maximum fold change seen across biomarkers. In order to facilitate comparison between p-tau measures, y-axes were scaled to the maximum fold change seen across biomarkers.

By comparison to all Aβ-negative participants, CSF p-tau levels were increased in Aβ-positive CU, MCI, and AD (Figure 4). In Aβ-positive MCI and AD, the mean fold increases (compared to Aβ-negative CU) were between 7.36 (95% CI, 6.26–8.47) and 13.27 (95% CI, 12.04–14.51) for p-tau217_Lilly_. By comparison, p-tau181_Lilly_ showed a mean fold increase of between 3.38 (95% CI, 2.98–3.79) and 5.35 (95% CI, 4.89–5.81), while p-tau231_ADx_ showed mean fold increases of between 2.40 (95% CI, 2.57–3.18) and 3.96 (95% CI, 3.68–4.25). Mean fold increases were between 1.72 (95% CI, 1.59–1.86) and 2.14 (95% CI, 1.99–2.30) for p-tau181_Innotest_ and between 1.88 (95% CI, 1.71–2.05) and 2.47 (95% CI, 2.27–2.67) for p-tau181_Elecsys_. In Aβ-positive CU, the greatest fold increase was seen for p-tau217_Lilly_ (4.78 [95% CI, 4.02–5.54), followed by p-tau181_Lilly_ (2.61 [95% CI, 2.29–2.92]) and p-tau231_ADx_ (2.39 [95% CI, 2.15–2.63]), p-tau181_Elecsys_ (1.66 [95% CI, 1.53–1.79]) and p-tau181_Innotest_ (1.58 [95% CI, 1.44–1.68]).

### Diagnostic Accuracy of CSF P-Tau Isoforms

**Figure 5:**
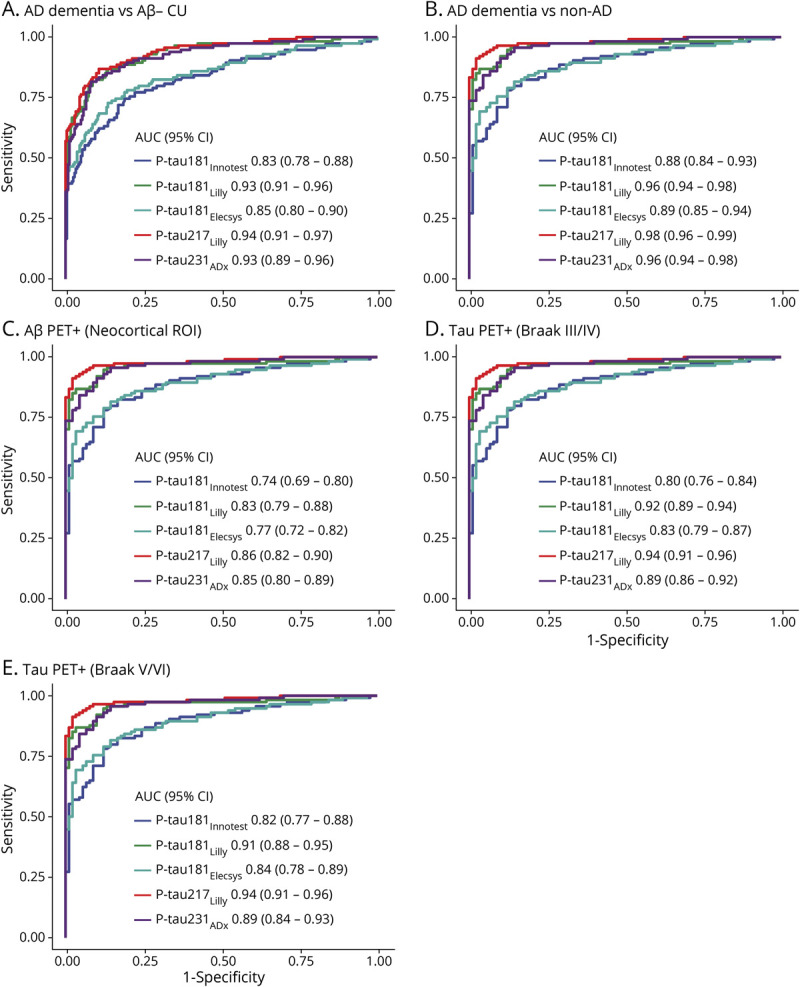
Receiver Operating Characteristic Plots for CSF Phosphorylated Tau (P-Tau) Receiver operating characteristic curves are shown for the following groups: Alzheimer disease (AD) dementia vs β-amyloid (Aβ)–negative cognitively unimpaired (CU) (A), AD dementia vs non-AD disorders (B), Aβ-PET positive vs negative (C), and tau-PET positive vs negative using the Braak III/IV (D) and V/VI (E) regions of interest (ROIs). AUC = area under the receiver operating characteristic curve; CI = confidence interval.

ROC curves and associated AUC values are shown in Figure 5. AUC values—along with sensitivity and specificity estimates at cutoffs that resulted in the highest Youden index—are reported in Table 2. The diagnostic performance of CSF p-tau for AD dementia vs Aβ-negative CU (Figure 5A) and non-AD neurodegenerative disorders (Figure 5B) was highest using p-tau217_Lilly_. For both contrasts, AUC values for p-tau217_Lilly_ were significantly higher than those for p-tau181_Innotest_ and p-tau181_Elecsys_ (*p* < 0.0001). For the separation of AD dementia from non-AD neurodegenerative disorders, the AUC value for p-tau217_Lilly_ was significantly higher than that for p-tau181_Lilly_ and p-tau231_ADx_ (*p* < 0.05). For both contrasts, AUC values for p-tau181_Lilly_ were significantly higher than those for p-tau181_Innotest_ and p-tau181_Elecsys_ (*p* < 0.0001).

**Table 2:**
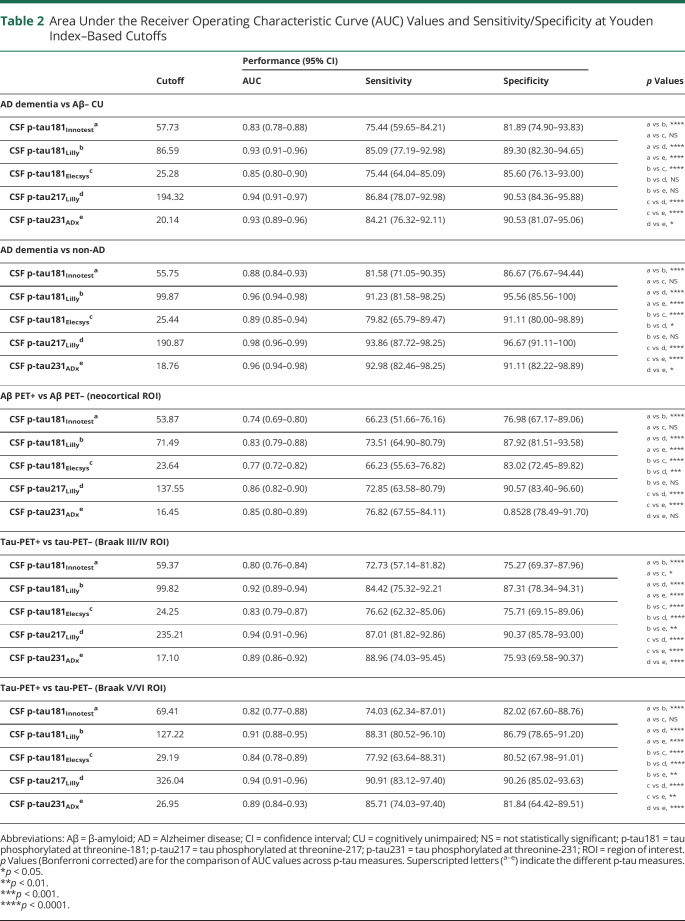
Area Under the Receiver Operating Characteristic Curve (AUC) Values and Sensitivity/Specificity at Youden Index–Based Cutoffs

When differentiating Aβ-PET–positive from Aβ-PET–negative participants (Figure 5C), p-tau217_Lilly_ outperformed p-tau181_Innotest_, p-tau181_Lilly_, and p-tau181_Elecsys_ (*p* < 0.0001). Using tau-PET status in the Braak III/IV (Figure 5D) and V/VI (Figure 5E) ROIs, AUCs for p-tau217_Lilly_ were significantly higher than those for p-tau181 and p-tau231_ADx_ (*p* < 0.0001). Using both Aβ and tau-PET, the diagnostic performance of p-tau181_Lilly_ was superior to that of p-tau181_Innotest_ and p-tau181_Elecsys_ (*p* < 0.0001). Using tau-PET, AUC values for p-tau231_ADx_ were significantly higher than those for p-tau181_Innotest_ (Braak III/IV and Braak V/VI, *p* < 0.0001), p-tau181_Elecsys_ (Braak III/IV, *p* < 0.0001; Braak V/VI, *p* < 0.01), and p-tau181_Lilly_ (Braak III/IV and Braak V/VI, *p* < 0.01). The AUC value of p-tau181_Elecsys_ was significantly higher than that for p-tau181_Innotest_ using the Braak III/IV ROI (*p* < 0.05) but not when using the Braak V/VI ROI.

## Discussion

Consistent with previous work using these assays, levels of CSF p-tau181_Lilly_ and p-tau217_Lilly_ were progressively higher across both the AD continuum (i.e., moving from Aβ-positive CU through Aβ-positive AD dementia)^14,15^ and tau-PET Braak stages.^14^ Furthermore, in agreement with a previous study, we found that p-tau217_Lilly_ had significantly higher correlations with Aβ and tau-PET as compared to p-tau181_Lilly_^14^ and extended this finding to show that the correlation was also significantly higher than for p-tau181_Innotest_, p-tau181_Elecsys_, and p-tau231_ADx_. Previously, using CSF samples taken prior to baseline tau-PET in Aβ-positive CU, 56% of patients showed positive p-tau217_Lilly_ levels, compared with only 25% for p-tau181_Lilly_.^15^ Combined with mass spectrometry findings in AD showing an increased degree of phosphorylation at threonine 217 compared with position 181,^16,34^ these results were interpreted as suggesting that phosphorylation at position 217 may be more pronounced by comparison to other sites. Although the differences were modest, stronger correlations observed with PET would also prove consistent with findings showing that threonine 217 phosphorylation was considerably increased in AD as compared to threonine 181^8,35^ and with the preferential phosphorylation of tau at specific sites across the different stages of AD.^16,36^ In addition, by comparison to studies using p-tau181 measurements from commercial assays such as p-tau181_Innotest_ and p-tau181_Elecsys_,^37^ larger effect sizes were seen when using p-tau181_Lilly_, p-tau217_Lilly_, and p-tau231_ADx_.

Using spline-based analyses, we compared the slopes of p-tau isoforms in relation to continuous Aβ and tau-PET SUVR values. These analyses were performed in CU individuals and in cognitively impaired Aβ-positive patients. Although greater PET SUVR values were associated with higher CSF p-tau concentrations for all isoforms, no significant differences were seen in the courses of p-tau181_Lilly_ and p-tau231_ADx_. By contrast, comparison of CIs showed that the slope of p-tau217_Lilly_ diverged from those of p-tau181_Lilly_ and p-tau231_ADx_ across a range of SUVR values, particularly in the CU group when using tau-PET in the Braak I/II ROI. These findings are consistent with increases in the active production of soluble tau in the presence of aggregated Aβ^8^ and, possibly, with the idea that the relative phosphorylation of tau at specific sites varies across the course of AD.^16,36^ Although findings with tau-PET in the cognitively impaired group suggest a plateau in the course of all 3 isoforms—possibly due to a process though which phosphorylation rates are reduced due to sequestration by hyperphosphorylated aggregates^38,39^—phosphorylation of threonine 217 may continue to increase later into the disease course, similar, for example, to what has been reported for p-tau205.^16^ This, combined with p-tau217_Lilly_ possibly showing a higher specificity for AD,^14^ may explain the higher AUC values seen for p-tau217_Lilly_. Although a tau-centric hypothesis ascribing a primary role to tau^40^ has been proposed as an alternative to the view that AD is caused by the accumulation of Aβ in the brain,^41^ both spline- and correlation-based sensitivity analyses in CU individuals by Aβ status showed there to be little association with Aβ and tau-PET in Aβ-negative CU individuals.

A recent study focused on characterizing the patterns of change in p-tau231_ADx_ and p-tau181_Elecsys_ in preclinical AD.^42^ In ROC analyses, the authors found that p-tau231_ADx_ had statistically significant higher predictive accuracies than p-tau181_Elecsys_ for discriminating Aβ-positive from Aβ-negative CU individuals. Moreover, p-tau231_ADx_ showed an AUC that was higher than that of p-tau181_Elecsys_. Although our findings showing that p-tau231_ADx_ had higher AUCs compared to p-tau181_Elecsys_ and p-tau181_Innotest_ are consistent with this, our results also suggest that p-tau231_ADx_ is similar to p-tau181_Lilly_. In a related study by Karikari et al.,^43^ N-p-tau217 showed higher diagnostic performance for identifying Aβ pathology and AD at the MCI stage compared to established p-tau181 assays (p-tau181_Innotest_ and p-tau181_Lumipulse_), but not compared to N-p-tau181. Possibly complicating this comparison, however, is the comparatively small number of prodromal AD cases. Although our results cannot be directly compared because of differences in the assays used for p-tau217, studies thus far suggest, overall, that p-tau217 assays are generally more sensitive. Further studies directly comparing these assays are required, however, as well as whether p-tau231_ADx_ and p-tau181_Lilly_ begin to increase at the same time point or if p-tau231_ADx_ starts to increase earlier in order to help establish the temporal dynamics of these different measures. Here, longitudinal studies comparing p-tau181, p-tau217, and p-tau231 will prove crucial.

Clinical utility in terms of fold change with respect to levels in Aβ-negative CU individuals varied across the investigated CSF p-tau measures. Although sharing the same p-tau181–specific antibody (AT270), the Innotest and Lilly p-tau181 assays had different total tau (i.e., not binding to the phosphorylation site) antibodies and showed large differences in fold change. This indicates the importance of tau isoforms or fragmentation with respect to clinical utility: should fragmentation occur in the region of the protein where the 2 total-tau antibodies bind, this could lead to the measurement of different pools of tau present in CSF. This hypothesis is reinforced by the fact that p-tau181_Innotest_ and p-tau181_Elecsys_ assays showed similar performance despite differing in both p-tau181–specific and total tau antibodies; presumably, this reflects these assays measuring the same tau isoform/fragment. The influence of the total tau antibody on the clinical utility of p-tau181 is thus significant, as p-tau181_Lilly_ showed about double the fold change as p-tau181_Innotest_ and p-tau181_Elecsys_ in AD. One explanation for the differences in fold change is also the possibility of a different binding affinity of the total tau antibody for tau, which could lead to differences in the measured signal with increasing protein concentrations. However, the Lilly assays showed that p-tau217 showed greater fold change compared to p-tau181 when using the same total tau antibody in combination with different phosphorylation-specific antibodies. The variability observed in the fold change of the measurements with p-tau181 assay cannot therefore be fully explained by technical differences of the assay or antibody affinity. One could speculate that the binding of antibodies to different phospho-epitopes could lead to conformational changes in the protein and therefore different affinity of the total tau antibody, but further studies are needed to demonstrate this. Similarly, we do not yet know whether p-tau231 will be better or worse in a head-to-head comparison to p-tau217 as the p-tau231_ADx_ assay uses a different total tau antibody.

Recent mass spectrometry–based work^44^ addressing tau PTMs has shown that p-tau181, p-tau217, and p-tau231 appear to be indicators of early AD pathology based on Braak NFT staging of postmortem brain tissue.^18^ In a related study exploring the biochemical link between measures of Aβ and tau phosphorylation, however, a somewhat different conclusion was reached: while soluble p-tau181, p-tau217, and p-tau231 were highly correlated to Aβ levels,^45^ the highest degree of tau phosphorylation was observed in the insoluble fractions of AD brain tissue, suggesting that correlations with Aβ and tau aggregates may be more complex than simple linear relations. Despite this recent progress in understanding the link between abnormal PTMs and the aggregation of tau in AD, additional studies are required to understand how such abnormal PTMs are reflected in predominantly C-terminally truncated tau.^8,46^ Current findings nevertheless highlight the importance of mapping PTMs in order to better understand the pathophysiology of AD; moreover, increased insight into the role of PTMs will facilitate the identification of novel therapeutic targets and improve AD diagnostics.

Strengths of our study include the large number of patients spanning the AD continuum, within-subject measurements of multiple CSF p-tau isoforms and their comparison to the widely used p-tau181 from Innotest and Elecsys, and the availability of Aβ and tau-PET imaging. Moreover, the use of mid-fragments across all p-tau measures allowed for a more direct comparison of p-tau biomarkers. This study has limitations, however. First, our inferences as to the ordering of changes in p-tau isoforms over the course of AD are based on cross-sectional data whereas longitudinal studies are needed to accurately address this question. Second, we did not have Aβ-PET in the AD dementia group. Although earlier work indicated that Aβ pathology may reach a plateau during the dementia phase of AD,^47^ recent findings suggest that this may not be the case.^48^ As such, we were not able to examine the effect of higher Aβ-PET SUVR values on p-tau isoforms but were nonetheless able to identify the significantly higher dynamic range of p-tau217_Lilly_ using the available Aβ-PET from participants with out dementia. Although we acknowledge the lack of Aβ-PET across all groups as a limitation, very high concordance is seen between CSF Aβ42/Aβ40 and Aβ-PET.^49^ As such, the 2 measures provide similar information with respect to defining Aβ status. As we were interested in the relationship between p-tau and the amount of fibrillary brain Aβ, however, we chose Aβ-PET as this measure reflects the cumulative burden of accumulated Aβ pathology while CSF Aβ42/40 reflects the production and clearance of Aβ42 and Aβ40 at a given time point.^50^ Lastly, though our study used the same assay for p-tau231_ADx_ and p-tau181_Elecsys_ as used in the study by Suárez-Calvet et al.,^42^ assays for p-tau181 and p-tau217 differed. In order to more definitively address the ordering of p-tau biomarkers, future work comparing phosphorylation epitopes will require the use of assays that are as similar as possible using head-to-head designs and validation in independent datasets.

We found that CSF p-tau217_Lilly_ more strongly correlated with Aβ and tau-PET and showed greater increases as compared to p-tau181_Innotest_, p-tau181_Lilly_, p-tau181_Elecsys_, and p-tau231_ADx_ in AD dementia and across tau-PET Braak stages. Moreover, CSF p-tau217_Lilly_ showed greater discriminative accuracy for AD dementia, as compared to CSF p-tau181_Innotest_, p-tau181_Lilly_, p-tau181_Elecsys_, and p-tau231_ADx_. These results suggest that CSF p-tau217_Lilly_ should be the preferred p-tau variant to use for AD diagnostics and for tracking disease progression (e.g., as an outcome in clinical AD trials).

## Glossary

Aβ: β-amyloid
AD: Alzheimer disease
AUC: area under the receiver operating characteristic curve
CBS: corticobasal syndrome
CI: confidence interval
CU: cognitively unimpaired
DSM-5: Diagnostic and Statistical Manual of Mental Disorders, 5th edition
FTD: frontotemporal dementia
MCI: mild cognitive impairment
MSD: Meso Scale Discovery
NFT: neurofibrillary tangle
p-tau: phosphorylated tau
p-tau181: tau phosphorylated at threonine-181
p-tau217: tau phosphorylated at threonine-217
p-tau231: tau phosphorylated at threonine-231
PD: Parkinson disease
PSP: progressive supranuclear palsy
PTM: post-translational modification
ROC: receiver operating characteristic
ROI: region of interest
SUVR: standardized uptake value ratio
VaD: vascular dementia

## References

[1] Grundke-Iqbal I, Iqbal K, Tung YC, Quinlan M, Wisniewski HM, Binder LI. Abnormal phosphorylation of the microtubule-associated protein tau (tau) in Alzheimer cytoskeletal pathology. Proc Natl Acad Sci USA. 1986;83(13):4913-4917. 3088567 10.1073/pnas.83.13.4913 PMC323854

[2] Petersen RC, Aisen P, Boeve BF, et al. Mild cognitive impairment due to Alzheimer disease in the community. Ann Neurol. 2013;74(2):199-208. 23686697 10.1002/ana.23931 PMC3804562

[3] Hansson O, Zetterberg H, Buchhave P, Londos E, Blennow K, Minthon L. Association between CSF biomarkers and incipient Alzheimer's disease in patients with mild cognitive impairment: a follow-up study. Lancet Neurol. 2006;5(3):228-234. 16488378 10.1016/S1474-4422(06)70355-6

[4] Skillback T, Farahmand T, Rosen C, et al. Cerebrospinal fluid tau and amyloid-beta1-42 in patients with dementia. Brain. 2015;138(pt 9):2716-2731. 26133663 10.1093/brain/awv181

[5] Barthélemy NR, Fenaille F, Hirtz C, et al. Tau protein quantification in human cerebrospinal fluid by targeted mass spectrometry at high sequence coverage provides insights into its primary structure heterogeneity. J Proteome Res. 2016;15(9):667-676. 26742856 10.1021/acs.jproteome.5b01001

[6] Cicognola C, Brinkmalm G, Wahlgren J, et al. Novel tau fragments in cerebrospinal fluid: relation to tangle pathology and cognitive decline in Alzheimer's disease. Acta Neuropathol. 2019;137(2):279-296. 30547227 10.1007/s00401-018-1948-2 PMC6514201

[7] Meredith JE Jr, Sankaranarayanan S, Guss V, et al. Characterization of novel CSF Tau and ptau biomarkers for Alzheimer's disease. PLoS One. 2013;8(10):e76523. 24116116 10.1371/journal.pone.0076523 PMC3792042

[8] Sato C, Barthélemy NR, Mawuenyega KG, et al. Tau kinetics in neurons and the human central nervous system. Neuron. 2018;97(6):1284-1298.e7. 29566794 10.1016/j.neuron.2018.02.015 PMC6137722

[9] Goedert M, Wischik CM, Crowther RA, Walker JE, Klug A. Cloning and sequencing of the cDNA encoding a core protein of the paired helical filament of Alzheimer disease: identification as the microtubule-associated protein tau. Proc Natl Acad Sci USA. 1988;85(11):4051-4055. 3131773 10.1073/pnas.85.11.4051 PMC280359

[10] Blennow K, Wallin A, Agren H, Spenger C, Siegfried J, Vanmechelen E. Tau protein in cerebrospinal fluid: a biochemical marker for axonal degeneration in Alzheimer disease? Mol Chem Neuropathol. 1995;26(3):231-245. 8748926 10.1007/BF02815140

[11] Vincent I, Zheng JH, Dickson DW, Kress Y, Davies P. Mitotic phosphoepitopes precede paired helical filaments in Alzheimer's disease. Neurobiol Aging. 1998;19(4:287-296. 9733160 10.1016/s0197-4580(98)00071-2

[12] Buerger K, Ewers M, Pirttilä T, et al. CSF phosphorylated tau protein correlates with neocortical neurofibrillary pathology in Alzheimer's disease. Brain. 2006;129(pt 11):3035-3041. 17012293 10.1093/brain/awl269

[13] Buerger K, Alafuzoff I, Ewers M, Pirttilä T, Zinkowski R, Hampel H. No correlation between CSF tau protein phosphorylated at threonine 181 with neocortical neurofibrillary pathology in Alzheimer's disease. Brain. 2007;130(pt 10):e82. 17615094 10.1093/brain/awm140

[14] Janelidze S, Stomrud E, Smith R, et al. Cerebrospinal fluid p-tau217 performs better than p-tau181 as a biomarker of Alzheimer's disease. Nat Commun. 2020;11(1):1683. 32246036 10.1038/s41467-020-15436-0 PMC7125218

[15] Mattsson-Carlgren N, Andersson E, Janelidze S, et al. Abeta deposition is associated with increases in soluble and phosphorylated tau that precede a positive tau PET in Alzheimer's disease. Sci Adv. 2020;6(16):eaaz2387. 32426454 10.1126/sciadv.aaz2387 PMC7159908

[16] Barthélemy NR, Li Y, Joseph-Mathurin N, et al. A soluble phosphorylated tau signature links tau, amyloid and the evolution of stages of dominantly inherited Alzheimer's disease. Nat Med. 2020;26(3):398-407. 32161412 10.1038/s41591-020-0781-z PMC7309367

[17] Jack CR Jr, Bennett DA, Blennow K, et al. NIA-AA Research Framework: toward a biological definition of Alzheimer's disease. Alzheimers Dement. 2018;14(4):535-562. 29653606 10.1016/j.jalz.2018.02.018 PMC5958625

[18] Wesseling H, Mair W, Kumar M, et al. Tau PTM profiles identify patient heterogeneity and stages of Alzheimer's disease. Cell. 2020;183(6):1699-1713.e13. 33188775 10.1016/j.cell.2020.10.029 PMC8168922

[19] American Psychiatric Association. Diagnostic and Statistical Manual of Mental Disorders: DSM-5. American Psychiatric Association; 2013.

[20] Armstrong MJ, Litvan I, Lang AE, et al. Criteria for the diagnosis of corticobasal degeneration. Neurology. 2013;80(5):496-503. 23359374 10.1212/WNL.0b013e31827f0fd1 PMC3590050

[21] Höglinger GU, Respondek G, Stamelou M, et al. Clinical diagnosis of progressive supranuclear palsy: the Movement Disorder Society criteria. Mov Disord. 2017;32(6):853-864. 28467028 10.1002/mds.26987 PMC5516529

[22] Postuma RB, Berg D, Adler CH, et al. The new definition and diagnostic criteria of Parkinson's disease. Lancet Neurol. 2016;15(6):546-548. 27302120 10.1016/S1474-4422(16)00116-2

[23] Rascovsky K, Hodges JR, Knopman D, et al. Sensitivity of revised diagnostic criteria for the behavioural variant of frontotemporal dementia. Brain. 2011;134(pt 9):2456-2477. 21810890 10.1093/brain/awr179 PMC3170532

[24] Román GC, Tatemichi TK, Erkinjuntti T, et al. Vascular dementia: diagnostic criteria for research studies: report of the NINDS-AIREN International Workshop. Neurology. 1993;43(2):250-260. 8094895 10.1212/wnl.43.2.250

[25] Leuzy A, Smith R, Ossenkoppele R, et al. Diagnostic performance of RO948 F 18 tau positron emission tomography in the differentiation of Alzheimer disease from other neurodegenerative disorders. JAMA Neurol. 2020;77(8):955-965. 32391858 10.1001/jamaneurol.2020.0989 PMC7215644

[26] Hansson O, Seibyl J, Stomrud E, et al. CSF biomarkers of Alzheimer's disease concord with amyloid-beta PET and predict clinical progression: a study of fully automated immunoassays in BioFINDER and ADNI cohorts. Alzheimers Dement. 2018;14(11):1470-1481. 29499171 10.1016/j.jalz.2018.01.010 PMC6119541

[27] Smith R, Scholl M, Leuzy A, et al. Head-to-head comparison of tau positron emission tomography tracers [F]flortaucipir and [F]RO948. Eur J Nucl Med Mol Imaging. 2020;47:342-354. 31612245 10.1007/s00259-019-04496-0 PMC6974501

[28] Palmqvist S, Zetterberg H, Blennow K, et al. Accuracy of brain amyloid detection in clinical practice using cerebrospinal fluid beta-amyloid 42: a cross-validation study against amyloid positron emission tomography. JAMA Neurol. 2014;71(10):1282-1289. 25155658 10.1001/jamaneurol.2014.1358

[29] Lundqvist R, Lilja J, Thomas BA, et al. Implementation and validation of an adaptive template registration method for 18F-flutemetamol imaging data. J Nucl Med. 2013;54(8):1472-1478. 23740104 10.2967/jnumed.112.115006

[30] Jack CR Jr, Wiste HJ, Weigand SD, et al. Defining imaging biomarker cut points for brain aging and Alzheimer's disease. Alzheimers Dement. 2017;13(3):205-216. 27697430 10.1016/j.jalz.2016.08.005 PMC5344738

[31] Cho H, Choi JY, Hwang MS, et al. In vivo cortical spreading pattern of tau and amyloid in the Alzheimer disease spectrum. Ann Neurol. 2016;80(2):247-258. 27323247 10.1002/ana.24711

[32] Diedenhofen B, Musch J. cocor: A comprehensive solution for the statistical comparison of correlations. PLoS One. 2015;10(3):e0121945. 25835001 10.1371/journal.pone.0121945 PMC4383486

[33] DeLong ER, DeLong DM, Clarke-Pearson DL. Comparing the areas under two or more correlated receiver operating characteristic curves: a nonparametric approach. Biometrics. 1988;44(3):837-845. 3203132

[34] Barthélemy NR, Mallipeddi N, Moiseyev P, Sato C, Bateman RJ. Tau phosphorylation rates measured by mass spectrometry differ in the intracellular brain vs. extracellular cerebrospinal fluid compartments and are differentially affected by Alzheimer's disease. Front Aging Neurosci. 2019;11:121. 31178717 10.3389/fnagi.2019.00121 PMC6537657

[35] Barthelemy NR, Bateman R, Marin P, et al. Tau hyperphosphorylation on T217 in cerebrospinal fluid is specifically associated to amyloid-β pathology. BioRxiv. Epub 2017 Nov 30.

[36] Medina M, Avila J. Further understanding of tau phosphorylation: implications for therapy. Expert Rev Neurother. 2015;15(1):115-122. 25555397 10.1586/14737175.2015.1000864

[37] Olsson B, Lautner R, Andreasson U, et al. CSF and blood biomarkers for the diagnosis of Alzheimer's disease: a systematic review and meta-analysis. Lancet Neurol. 2016;15:673-684. 27068280 10.1016/S1474-4422(16)00070-3

[38] Yanamandra K, Patel TK, Jiang H, et al. Anti-tau antibody administration increases plasma tau in transgenic mice and patients with tauopathy. Sci Transl Med. 2017;9(386):eaal2029. 28424326 10.1126/scitranslmed.aal2029 PMC5727571

[39] McDade E, Wang G, Gordon BA, et al. Longitudinal cognitive and biomarker changes in dominantly inherited Alzheimer disease. Neurology. 2018;91(14):e1295-e1306. 30217935 10.1212/WNL.0000000000006277 PMC6177272

[40] Braak H, Del Tredici K. Amyloid-beta may be released from non-junctional varicosities of axons generated from abnormal tau-containing brainstem nuclei in sporadic Alzheimer's disease: a hypothesis. Acta Neuropathol. 2013;126(2):303-306. 23824268 10.1007/s00401-013-1153-2

[41] Hardy J, Allsop D. Amyloid deposition as the central event in the aetiology of Alzheimer's disease. Trends Pharmacol Sci. 1991;12(10):383-388. 1763432 10.1016/0165-6147(91)90609-v

[42] Suárez-Calvet M, Karikari TK, Ashton NJ, et al. Novel tau biomarkers phosphorylated at T181, T217 or T231 rise in the initial stages of the preclinical Alzheimer's continuum when only subtle changes in Abeta pathology are detected. EMBO Mol Med. 2020;12(12):e12921. 33169916 10.15252/emmm.202012921 PMC7721364

[43] Karikari TK, Emersic A, Vrillon A, et al. Head-to-head comparison of clinical performance of CSF phospho-tau T181 and T217 biomarkers for Alzheimer's disease diagnosis. Alzheimers Dement. 2020:17(5):755-767. 33252199 10.1002/alz.12236 PMC8246793

[44] Mair W, Muntel J, Tepper K, et al. FLEXITau: quantifying post-translational modifications of tau protein in vitro and in human disease. Anal Chem. 2016;88(7):3704-3714. 26877193 10.1021/acs.analchem.5b04509 PMC5808556

[45] Horie K, Barthélemy NR, Mallipeddi N, et al. Regional correlation of biochemical measures of amyloid and tau phosphorylation in the brain. Acta Neuropathol Commun. 2020;8(1):149. 32854776 10.1186/s40478-020-01019-z PMC7450927

[46] Kanmert D, Cantlon A, Muratore CR, et al. C-terminally truncated forms of tau, but not full-length tau or its C-terminal fragments, are released from neurons independently of cell death. J Neurosci. 2015;35(30):10851-10865. 26224867 10.1523/JNEUROSCI.0387-15.2015 PMC6605107

[47] Villemagne VL, Pike KE, Chetelat G, et al. Longitudinal assessment of Abeta and cognition in aging and Alzheimer disease. Ann Neurol. 2011;69(1):181-192. 21280088 10.1002/ana.22248 PMC3045039

[48] Rullmann M, McLeod A, Grothe M, Sabri O, Barthel H. Reshaping the amyloid buildup curve in Alzheimer's disease?—partial volume effect correction of longitudinal amyloid PET data. J Nucl Med. 2020;61(12):1820-1824. 32358089 10.2967/jnumed.119.238477 PMC9364900

[49] Blennow K, Mattsson N, Schöll M, Hansson O, Zetterberg H. Amyloid biomarkers in Alzheimer's disease. Trends Pharmacol Sci. 2015;36(5):297-309. 25840462 10.1016/j.tips.2015.03.002

[50] Blennow K, Hampel H. CSF markers for incipient Alzheimer's disease. Lancet Neurol. 2003;2(10):605-613. 14505582 10.1016/s1474-4422(03)00530-1

